# Correspondence

**Published:** 1870-03

**Authors:** F. K. Bailey

**Affiliations:** Knoxville, Tenn.


KNOXVILLE, TENN., *Feb., 1870.*

PROFESSOR DAVIS:—The weather here during the last summer was hotter than usual and uncommonly dry.

During the hottest week the mercury stood as follows:—

| | | | | |
|---|---|---|---|---|
| Tuesday, | August | 17th, | 3 P.M., | 97 |
| Wednesday, | " | 18th, | 3 " | 99½ |
| " | " | " | 4 " | 101 |
| Thursday, | " | 19th, | 3 " | 98 |
| " | " | " | 4 " | 100 |
| Friday, | " | 20th, | 3 " | 100 |
| Saturday, | " | 21st, | 1½ " | 101 |
| " | " | " | 3 " | 102 |
| " | " | " | 4 " | 100 |

Excepting summer complaint among children, there was but little sickness during the summer, and the autumn months were unusually healthy. There have been several deaths from disease of the lungs, during the last nine months. Two of the victims were young men, who were healthy till since the war. They are the only cases occurring among the white population. It is among the blacks that pulmonary disease occurs most frequently, and many are the victims. In one family, three sisters have died since May last. The first was married and mother of one child. She began to cough in February, and in March had measles. When I first saw her, there was rapid softening at the lower portion of the left lung. There was pleuritic inflammation, which rendered it more painful; and from week to week, it was easy to trace the progress of the disorganization upwards. Large quantities of muco-purulent sputum was expectorated every morning, and dyspnœa was so distressing that she could not lie down at all. It seemed as if the morbid secretion was so abundant as to press constantly upon the bronchial tubes and cause suffocation, when she was in a recumbent posture. She died in May, and fully two-thirds of the lung had become destroyed. The second sister died in summer, with a less extent of disease, but of the same character.

The third was but 16 years old. She had measles in early spring and began to cough at once. In April, my attention was particularly called to a diarrhœa which was difficult to control. The cough continued, and both lungs were diseased. There had also been suppression of the menses since January. I did not visit her regularly, but saw her at times, while attending upon other patients in the vicinity. In July, was called on account of an indolent swelling of the right mamma, and found that she was pregnant.

The poor girl gave a look of injured innocence when I called her attention to the rotundity and increased size in the hypogastrium, and suggested that in her social relations she had been somewhat erratic. Emaciation had increased, and her cough was incessant. In September, I was called to attend her in labor, but other engagements prevented. I learned that she had a child, which is now living, but she died in December. I relate the above cases to show how rapidly the colored race is vanishing away. They were mulattoes, and their mother is full-blooded African. Their paternity must have been nearly, if not wholly Anglo-Saxon.

The unmixed blacks seldom have consumption, but frequently die of acute pneumonia. I am inclined to think that at least half of the children die before they are three years old. Very many are of uncertain parentage, which fact may account for the high rate of mortality.

There is a want of true parental affection, which we all know is necessary, in order that the wants of helpless infants may be anticipated and supplied. There are many questions naturally suggested in contemplating the condition, both present and future, of a race so recently released from bondage to freedom, which should interest the social and political economist. Matters of more vital importance to the colored race than the elective franchise will soon be crowded upon public consideration.

A case of tetanus occurred here last summer. I did not see the patient, and the following details were given to me by Dr. A. S. Nichole, who was in attendance in connection with others.

*July 6th, 1869.* Thomas Steele, aged 24, American, and acting as brakeman on the E. Tennessee and Georgia Railroad, was injured by being thrown from a car.

The lesion was a flesh wound or contusion upon the outer aspect of the left lower extremity, extending from the middle third of the thigh to a point three inches below the knee. One small artery was torn across, requiring ligation. Bones uninjured, and the articulation intact. The first dressings were adhesive straps and cold water. On the 12th, it was noticed that as the dressings were changed, there was œdema in the extremity, below the point of injury.

Granulations appeared healthy. On the 15th, twitching of the tensor muscles commenced, giving great pain, at intervals of two or three minutes. At this time, there was no difficulty in deglutition and no stiffness of the neck. Bromide potassii and brom. ammonia were given with no effect. Whiskey and beef-tea administered; morphine, hypodermically, at short intervals.

On the night of the 17th, complained of feeling tired, while eating. *18th.* Difficulty of protruding the tongue and opening the jaws. Dysphagia, with spasm of the whole body, at each accession of pain in the limb. Spasms opisthotonic, and muscles of abdomen tense. Aconite internally and externally administered.

*19th.* Dysphagia increasing. Bromide potassii given freely, with ergot, to diminish congestion of the spinal cord, as suggested by Brown-Séquard. Calabar bean could not be obtained. Death occurred on the 22d.

The weather, thus far, this winter, has been mild. The mercury one morning in first week in January went down to 14° above. It is seldom that we find it below 32° The principal diseases have been those of a catarrhal character. It is common to find a person complaining of pain in the frontal region, attended with irritation of the nasal and pharyngeal mucous membranes, gradually invading the larynx and bronchial tubes. When there is much febrile disturbance, the functions of the kidneys and skin are deranged, the secretions being very scanty. Active anti phlogistic treatment is called for. Absence of local malarious influence renders tonics seldom called for.

The effects of a short residence in a malarial region are shown in the following:—The parents of one of the infants alluded to in my remarks upon the bromides, in the January EXAMINER, removed in October to Southern Illinois. The change upon the child was favorable, as it soon became very fleshy.

After a stay of two months they concluded to return.

They had been here about one month when both mother and child had all the characteristic symptoms of intermittent. The chills were well-defined in the child, and entire freedom from fever after the usual time. It was only upon the recurrence of a second chill that its malarial character was suggested; and a few grains of quinine soon effected a cure.

The mother had a decided chill, with fever following, and attended with the soreness upon the lips.

Quinine was given, and no return of chills occurred. This was her first visit to a malarial region, being a native of this State. The father had lived in the North-West till the war, and, consequently, was not so susceptible.

But I have exceeded already my design in the length of this communication.

F. K. BAILEY, M.D.

